# The influence of sex and body mass index on the association between soluble neprilysin and risk of heart failure hospitalizations

**DOI:** 10.1038/s41598-021-85490-1

**Published:** 2021-03-15

**Authors:** Julio Núñez, Eduardo Núñez, Elena Revuelta-López, Gema Miñana, Jaume Barallat, Vicent Bodi, Juan Sanchis, Alberto Aimo, Michele Emdin, Josep Lupón, Oliver Husser, Antoni Bayes-Genis

**Affiliations:** 1 Cardiology Department, Hospital Clínico Universitario, INCLIVA, Departamento de Medicina, Universitat de València, Avda. Blasco Ibáñez 17, 46010 Valencia, Spain; 2 CIBER Cardiovascular, Madrid, Spain; 3 Biochemistry Department, Hospital Universitari Germans Trias i Pujol, Badalona, Spain; 4 Institute of Life Sciences, Scuola Superiore Sant’Anna, Pisa, Italy; 5 Cardiology Division, University Hospital of Pisa, Pisa, Italy; 6 Scuola Superiore Sant’Anna, Pisa, Italy; 7 Heart Failure Unit, Cardiology Department, Hospital Universitari Germans Trias i Pujol, Badalona, Spain; 8 Department of Medicine, Autonomous University of Barcelona, Barcelona, Spain; 9 Klinik für Innere Medizin I, Kardiologie, St. Johannes-Hospital Dortmund, Dortmund, Germany

**Keywords:** Cardiology, Biomarkers

## Abstract

A higher neprilysin activity has been suggested in women. In this retrospective analysis, we evaluated the association of sex and body mass index (BMI) with soluble neprilysin (sNEP) and recurrent admissions among 1021 consecutive HF outpatients. The primary and secondary endpoints were the number of HF hospitalizations and all-cause mortality, respectively. The association between sNEP with either endpoint was evaluated across sex and BMI categories (≥ 25 kg/m^2^ vs. < 25 kg/m^2^). Bivariate count regression (Poisson) was used, and risk estimates were expressed as incidence rates ratio (IRR). During a median follow-up of 6.65 years (percentile 25%-percentile 75%:2.83–10.25), 702 (68.76%) patients died, and 406 (40%) had at least 1 HF hospitalization. Median values of sNEP and BMI were 0.64 ng/mL (0.39–1.22), and 26.9 kg/m^2^ (24.3–30.4), respectively. Left ventricle ejection fraction was < 40% in 78.9% of patients, and 28% were women. In multivariable analysis, sNEP (main effect) was positively associated with HF hospitalizations (*p* = 0.001) but not with mortality (*p* = 0.241). The predictive value of sNEP for HF hospitalizations varied non-linearly across sex and BMI categories (*p*-value for interaction = 0.003), with significant and positive effect only on women with BMI ≥ 25 kg/m^2^ (*p* = 0.039). For instance, compared to men, women with sNEP of 1.22 ng/mL (percentile 75%) showed a significantly increased risk (IRRs: 1.26; 95% CI: 1.05–1.53). The interaction analysis for mortality did not support a differential prognostic effect for sNEP (*p* = 0.072). In conclusion, higher sNEP levels in overweight women better predicted an increased risk of HF hospitalization.

## Introduction

The enzyme neprilysin breaks down various vasoactive peptides, including natriuretic peptides, and plays a key role in the pathophysiology of heart failure (HF)^1^. Over the last few years, neprilysin inhibition has become a therapeutic target in patients with HF^2,3^. Subgroup analyses from randomized clinical trials indicate that neprilysin inhibition by sacubitril/valsartan may decrease the risk of recurrent hospitalization in women with HF with preserved ejection fraction^4,5^. Although some authors suggest that neprilysin activity is higher in women with HF^6,7^, the evidence explaining the sex-differential findings remains mostly speculative.

The circulating soluble form of the extracellular domain of neprilysin (sNEP) has emerged as a promising biomarker in patients with acute and chronic HF for predicting cardiovascular death and hospitalizations^8–11^, despite conflicting results between groups and different immunoassay utilized^12,13^. It is currently unclear whether the association between sNEP and hospitalization burden is modified by sex and body mass index (BMI). Therefore, we evaluated the influence of sex and BMI on the prognostic value of sNEP in patients with HF.

## Methods

### Study population

This is an observational retrospective analysis from a prospective registry carried on in a third-level university hospital^11^. We analyzed 1021 consecutive ambulatory patients evaluated from May 2006 to May 2013 at a multidisciplinary HF clinic. Data on patient demographics, medical history, physical examination, 12-lead electrocardiogram, laboratory tests, echocardiogram, and medications were included in pre-established electronic questionnaires. The principal referral criterion was HF diagnosis, according to the European Society of Cardiology guidelines^3^, with at least one hospitalization for acute heart failure (AHF) or reduced left ventricular ejection fraction (LVEF). No patient was on sacubitril/valsartan or other NEP inhibitors.

BMI was calculated in all patients using calibrated scales. The associations between sNEP and the outcomes were evaluated in the whole population and stratified patients according to sex and BMI (considering the standard cut-off of 25 kg/m^2^ used to categorize normal weight from overweight)^14^.

All participants provided written informed consent, and the local ethics committee (*Comité d’ètica de la Investigació Clínica del Hospital Universitari Germans Trias i Pujol*) approved the study. The study protocol conformed to the ethical guidelines of the 1975 Declaration of Helsinki (revised in 1983), as reflected by an a priori approval by the institution's human research committee. Patients were not involved in the design and conduct of this research.

### sNEP measurement

All blood samples were drawn between 09:00 a.m. and noon and stored as plasma at − 80 °C, without previous freeze–thaw cycles. We measured sNEP by a modified sandwich immunoassay (human neprilysin/CD10 ELISA kit, Aviscera Bioscience, Santa Clara, California, code No. SK00724-01, lot No. 20111893). To improve the analytical sensitivity of the method and obtain a lower limit of sample quantification, several modifications were made: serum aliquots were diluted one-quarter before incubation in dilution buffer provided by the manufacturer (DB09); the kit was transferred to an automated robotic platform (Basic Radim Immunoassay Operator 2 [BRIO 2], Radim SpA, Pomezia, Italy) that performed all incubations at a constant temperature of 30 °C, with 1,000 revolutions/min mixing; and the initial sample incubation was extended to 150 min to achieve a higher slope in the calibration curve and better assay sensitivity.

The assay measures the 52 to a 750-amino-acid fraction of neprilysin as an immunogen (extracellular soluble fraction). This assay has 0% cross-reactivity with the two metallopeptidases most similar to this sequence, endothelin converting enzymes 1 and 2. The test also does not exhibit cross-reactivity with erythrocyte cell-surface antigen (KELL), another protein with strong homology with neprilysin. The modified protocol presented analytical linearity from 0.250 to 4 ng/mL. Samples with concentrations > 4 ng/mL were further diluted to a final range of 0.250 to 64 ng/mL. At a positive control value of 1.4 ng/mL, the intra-assay and inter-assay coefficients of variation were 3.7% and 8.9%, respectively. The intra-assay coefficient of variation at the median value (0.642 ng/mL) was 6.5%.

### Follow-up and outcomes

We followed up all patients at regular predefined intervals, with additional visits when medically necessary^11^. These scheduled visits included, at a minimum, quarterly visits with nurses, biannual visits with physicians, and elective visits with geriatricians, psychiatrists, and rehabilitation physicians. Patients who did not attend the regular visits were contacted by telephone^11^. We selected the total number of unplanned HF-related hospitalizations as the primary endpoint. We identified hospitalizations from patients' clinical records in the HF unit and hospital wards through the electronic Catalan medical record database. All-cause mortality was selected as a secondary endpoint. We identified fatal events from the HF unit's clinical records, hospital wards, emergency room, general practitioners and by contacting the patients' relatives. We verified the reliability of the data by double-checking the databases of the Catalan and Spanish health systems. For this study, the personnel in charge of clinical management and endpoint adjudication were unaware of the patient's sNEP level.

### Statistical analysis

Continuous variables are expressed as mean ± standard deviation (SD) or median [percentile 25% (p25%) to percentile 75% (p75%)] per variable distribution. Discrete variables are presented as percentages. Baseline characteristics based on sex and BMI were compared by ANOVA, Kruskal–Wallis, or chi-squared tests, as appropriate. Rates of events were presented as per 1 person-years (P-Y). To account for the positive correlation between HF hospitalization and mortality, we fitted the Famoye bivariate Poisson regression model, where the number of admissions (as counts) and mortality (as the terminal event) were modeled simultaneously and linked by shared frailty^15^. To account for differences in each recurrent event, the log of follow-up time was included as an offset in each submodel. Crude and adjusted rates (number of events per 1 person-year) are presented among the groups tested. We selected explanatory variables for the initial multivariable model based on subject-matter knowledge. Then, using a backward elimination (BE) procedure which includes a fractional polynomial (FP) transformation for continuous variables^14^, we arrived to a final model. In some instances, however, the automatic selection procedure was overridden by leaving important known predictors in HF's setting independent of their level of significance. For HF hospitalization, the best sNEP polynomial transformation was FP[-0.5]. The sNEP trajectories across sex/BMI categories are depicted in graphs after back-transformation to its original scale. The final covariates (and its FP transformation in case of continuous variables) included in the HF-rehospitalization model were age (FP:1), HF duration (FP:1), New York Heart Association (NYHA) class III versus I–II, ischaemic heart disease, systolic blood pressure (FP:1), heart rate (FP:2), serum sodium (FP:3), N-terminal pro-B-type natriuretic peptide (NT-proBNP) (FP:0), estimated glomerular filtration rate [Modification of Diet in Renal Disease (MDRD) formula] (FP:1), high-sensitivity troponin T (FP:0), LVEF < 40% versus ≥ 40%, and treatment with angiotensin-converting enzyme inhibitors (ACEI) or angiotensin II receptor blockers (ARB), beta-blockers, mineralocorticoid receptor antagonists (MRA), or loop diuretics. For all-cause mortality, the covariates were the same, plus the inclusion of hemoglobin (FP:1) and ST2 (FP:-0.5). Risk estimates are presented as incidence rate ratios (IRRs). We set a two-sided *p* < 0.05 as the threshold for significance. All analyses were performed in Stata 15.1 (Stata Statistical Software, Release 15 [2017]; StataCorp LP, College Station, TX, USA). We used the "Bivcnto" Stata module for multivariable bivariate Poisson analyses.

## Results

### Baseline characteristics

The mean age was 66 ± 13 years, and 286 (28%) patients were women. Most of the patients had LVEF < 40% (78.9%), NYHA class I–II (75.7%), and ischaemic aetiology (51%). The median levels of sNEP and NT-proBNP were 0.64 ng/mL (p25%-p75%: 0.39–1.22) and 1248 ng/L (IQI: 538–2825), respectively. The median BMI was 26.9 kg/m^2^ (p25%-p75%: 24.4–30.4), and 701 (68.7%) patients had a BMI ≥ 25 kg/m^2^, with a similar distribution in both sexes (women vs. men: 69.9% vs. 68.2%, *p* = 0.585). The number and proportion of patients in the pre-specified categories were as follows: women/BMI < 25 kg/m^2^, n = 86 (8.4%); women/BMI ≥ 25 kg/m^2^, n = 200 (19.6%); men/BMI < 25 kg/m^2^, n = 234 (22.9%); and men/ BMI ≥ 25 kg/m^2^, n = 501 (49.1%). The baseline characteristics across the sex/BMI subgroups are presented in Table 1. Overall, overweight women showed higher prior hypertension, type 2 diabetes, and lower values of NT-proBNP (Table 1).

**Table 1:** Baseline characteristics across sex and BMI.

Variables	Women, BMI < 25 kg/m^2^ (n = 86)	Women, BMI ≥ 25 kg/m^2^ (n = 200)	Men, BMI < 25 kg/m^2^ (n = 234)	Men, BMI ≥ 25 kg/m^2^ (n = 501)	*p*-value
**Demographics and medical history**					
Age, years	69 ± 14	70 ± 10	67 ± 14	64 ± 12	< 0.01
BMI, kg/m^2^*	22.9 (21.2, 24.2)	29.9 (27.1, 33.8)	23.1 (21.6, 24.2)	28.6 (26.7, 31.2)	< 0.01
Hypertension, n (%)	52 (60.5)	153 (76.5)	125 (53.4)	307 (61.3)	< 0.01
DM, n (%)	24 (27.9)	90 (45.0)	59 (25.2)	187 (37.3)	< 0.01
Dyslipidemia, n (%)	31 (33.0)	88 (44.0)	96 (41)	266 (53.1)	< 0.01
Current smoker, n (%)	8 (9.3)	8 (4.0)	49 (20.9)	98 (19.6)	< 0.01
Former smoker, n (%)	9 (10.5)	15 (7.5)	126 (53.8)	276 (55.1)	< 0.01
COPD, n (%)	4 (4.6)	21 (10.5)	45 (19.2)	104 (20.9)	< 0.01
Ischemic etiology, n (%)	26 (30.27)	75 (37.5)	134 (57.3)	286 (57.1)	< 0.01
HF duration, months*	24 (2, 72)	24.3 (6, 70.3)	17.2 (2, 60)	28 (4, 72)	< 0.01
ICD, n (%)	5 (5.8)	17 (8.5)	31 (13.2)	83 (16.6)	< 0.01
CRT, n (%)	4 (4.5)	14 (7.0)	16 (6.8)	51 (10.2)	0.178
NYHA class I or II, n (%)	57 (66.3)	133 (66.5)	177 (75.6)	406 (81.0)	< 0.01
LVEF < 40%, n (%)	54 (62.8)	124 (62.0)	197 (84.2)	431 (86.0)	< 0.01
**Vital signs**					
Heart rate, bpm	75 ± 17	75 ± 14	71 ± 14	71 ± 14	0.07
SBP, mmHg	126 ± 23	132 ± 25	125 ± 24	126 ± 20	< 0.01
**Laboratory**					
Haemoglobin, g/dL	12.0 ± 1.7	12.0 ± 1.5	13.1 ± 2.0	13.2 ± 1.9	< 0.01
eGFR (MDRD), mL/min/1.73 m^2^	53 ± 29	53 ± 28	58 ± 29	62 ± 30	0.44
Serum sodium, mEq/L*	139 (137, 141)	140 (137, 142)	139 (136, 141)	139 (137, 141)	0.18
NT-proBNP, pg/mL*	2111 (1093, 6013)	988 (491, 2460)	1833 (707, 4008)	1055 (419, 2147)	< 0.01
Neprilysin, ng/mL *	1.39 (0.74, 2.30)	1.51 (0.74, 2.73)	1.59 (0.96, 3.14)	1.56 (1.82, 2.40)	0.16
**Medical treatment**					
Loop diuretics, n (%)	76 (88.4)	196 (98.0)	202 (86.3)	456 (91.0)	< 0.01
MRA, n (%)	51 (59.3)	115 (57.5)	120 (51.3)	311 (62.1)	0.05
Beta-blockers, n (%)	78 (90.7)	170 (85.0)	205 (87.6)	470 (93.8)	< 0.01
ACEI or ARB, n (%)	71 (82.6)	175 (87.5)	204 (87.2)	468 (93.4)	< 0.01

Continuous variables are expressed as mean ± 1 standard deviation unless otherwise specified.

*ACEI* angiotensin-converting enzyme inhibitors, *ARB* angiotensin II receptor blockers, *BMI* body mass index, *COPD* chronic obstructive pulmonary disease, *CRT* cardiac resynchronization therapy, *DM* diabetes mellitus, *eGFR* estimated glomerular filtration rate, *HF* heart failure, *ICD* implantable cardioverter-defibrillator, *LVEF* left ventricle ejection fraction, *MDRD* Modification of Diet in Renal Disease formula, *MRA* mineralocorticoid receptor antagonist, *NT-proBNP* amino-terminal pro-brain natriuretic peptide, *NYHA* New York Heart Association, *SBP* systolic blood pressure, *sNEP* soluble form of neprilysin.

*Variable expressed as median (interquartile interval).

### sNEP and baseline characteristics

Table 2 summarizes the baseline characteristics stratified by sNEP quintiles. Age, history of hypertension, ischaemic etiology, and LVEF < 40% were inversely associated with the sNEP quintile. In contrast, heart rate was positively associated with the sNEP quintile. We found no significant differences in sex, BMI, HF duration, NYHA functional class, systolic blood pressure, hemoglobin, sodium, renal function markers, or NT-proBNP among sNEP quartiles.

**Table 2:** Baseline characteristics across sNEP quintiles.

Variables	sNEP-Q1 (N = 205)	sNEP-Q2 (N = 204)	sNEP-Q3 (N = 204)	sNEP-Q4 (N = 204)	sNEP-Q5 (N = 204)	*p*-value
**Demographics and medical history**						
Age, years	69 ± 11	68 ± 11	67 ± 13	64 ± 14	64 ± 14	< 0.01
Men, n (%)	148 (72.2)	154 (75.5)	143 (70.1)	148 (72.5)	142 (69.6)	0.69
BMI, kg/m^2^	27.39 ± 5.90	27.97 ± 5.24	27.68 ± 4.95	27.77 ± 5.50	27.65 ± 5.27	0.87
Hypertension, n (%)	138 (67.3)	132 (64.7)	136 (66.7)	113 (55.4)	118 (57.8)	0.04
DM, n (%)	70 (34.1)	67 (32.8)	80 (39.2)	68 (33.3)	75 (36.8)	0.63
Dyslipidemia, n (%)	99 (48.3)	99 (48.5)	93 (45.6)	96 (47.1)	94 (46.1)	0.97
Current smoker, n (%)	28 (13.7)	39 (19.1)	27 (13.2)	43 (21.1)	26 (12.7)	0.06
Former smoker, n (%)	85 (41.5)	88 (43.1)	88 (43.1)	85 (41.7)	80 (39.2)	0.93
COPD, n (%)	30 (14.6)	48 (23.5)	32 (15.7)	29 (14.2)	35 (17.2)	0.08
IHD etiology, n (%)	124 (60.5)	101 (49.5)	105 (51.5)	96 (47.1)	95 (46.6)	0.03
HF duration, months	44.5 ± 58.0	51.4 ± 68.7	45.4 ± 60.9	48.6 ± 62.8	50.4 ± 63.2	0.75
ICD, n (%)	34 (16.6)	19 (9.3)	25 (12.3)	27 (13.2)	31 (15.2)	0.23
CRT, n (%)	23 (11.2)	10 (4.9)	20 (9.8)	12 (5.9)	20 (9.8)	0.09
NYHA class I or II, n (%)	163 (75.5)	152 (74.5)	150 (73.5)	158 (77.4)	150 (73.5)	0.531
LVEF < 40%, n (%)	164 (80.0)	170 (83.3)	171 (83.8)	154 (75.5)	147 (72.1)	0.013
**Vital signs**						
Heart rate, bpm	69 ± 12	72 ± 14	73 ± 14	73 ± 16	73 ± 15	0.04
SBP, mmHg	127 ± 25	125 ± 20	126 ± 20	128 ± 24	127 ± 23	0.73
**Laboratory**						
Hemoglobin, g/dL	12.7 ± 1.7	13.0 ± 1.9	12.7 ± 1.9	13.1 ± 2.0	12.9 ± 1.8	0.08
eGFR (MDRD), mL/min/1.73 m^2^	59 ± 27	60 ± 31	56 ± 29	59 ± 34	59 ± 26	0.79
eGFR < 60 mL/min/1.73 m^2^, n (%)	117 (57.1)	108 (52.9)	125 (61.3)	111 (54.4)	113 (55.4)	0.49
Serum sodium, mEq/L	139 ± 3	139 ± 4	139 ± 3	139 ± 3	139 ± 3	0.16
hsTNT, ng/L*	28 (14, 40)	22 (13, 37)	25 (11, 45)	26 (13, 43)	25 (12, 38)	0.64
NT-proBNP, pg/mL*	1310 (594, 2858)	1034 (540, 2635)	1318 (489, 2967)	1484 (550, 3114)	1165 (535, 2725)	0.73
Neprilysin, ng/mL*	0.25 (0.25, 0.26)	0.46 (0.39, 0.51)	0.64 (0.60, 0.70)	0.98 (0.84, 1.22)	3.25 (2.00, 8.92)	< 0.01
**Medical treatment**						
Loop diuretics, n (%)	184 (89.8)	191 (93.6)	188 (92.2)	181 (88.7)	186 (91.2)	0.44
MRA, n (%)	119 (58.0)	107 (52.5)	122 (59.8)	123 (60.3)	126 (61.8)	0.36
ACEI or ARB, n (%)	61 (29.8)	43 (21.1)	59 (28.9)	55 (27.0)	53 (26.0)	0.30
Beta-blockers, n (%)	187 (91.2)	189 (92.6)	183 (89.7)	180 (88.2)	179 (87.7)	0.44

Continuous variables are expressed as mean ± 1 standard deviation unless otherwise specified.

*ACEI* angiotensin-converting enzyme inhibitors, *ARB* angiotensin II receptor blockers, *BMI* body mass index, *COPD* chronic obstructive pulmonary disease, *CRT* cardiac resynchronization therapy, *DM* diabetes mellitus, *eGFR* estimated glomerular filtration rate, *HF* heart failure, *ICD* implantable cardioverter-defibrillator, *LVEF* left ventricle ejection fraction, *MDRD* Modification of Diet in Renal Disease formula, *MRA* mineralocorticoid receptor antagonist, *NT-proBNP* amino-terminal pro-brain natriuretic peptide, *NYHA* New York Heart Association, *SBP* systolic blood pressure, *sNEP* soluble form of neprilysin.

*Variable expressed as median (interquartile interval).

The median sNEP did not differ between women and men [0.68 ng/mL (p25%–p75%: 0.41–1.35), vs. 0.63 ng/mL (IQI: 0.39–1.19), *p* = 0.377], and BMI ≥ 25 kg/m^2^ versus < 25 kg/m^2^ [0.64 ng/mL (IQI: 0.41–1.23) vs. 0.65 ng/mL (IQI: 0.36–1.1), *p* = 0.560). Similarly, the sNEP distribution did not differ across sex and BMI categories [women/BMI < 25 kg/m^2^: 0.72 ng/mL (IQI: 0.44–134); women/BMI ≥ 25 kg/m^2^: 0.66 ng/mL (IQI: 0.37–1.35); men/BMI < 25 kg/m^2^: 0.63 ng/mL (IQI: 0.32–1.04); men/BMI ≥ 25 kg/m^2^: 0.64 ng/mL (IQI: 0.42–1.22); *p* = 0.297].

### Follow-up

Over a median follow-up of 6.65 years [p25%–p75%:2.83–10.25], 702 (68.76%) patients died. A total of 959 HF-hospitalizations were recorded in 406 patients (40%). Of these, 231 patients (56.9%) had two or more admissions.

### HF hospitalization risk and sex/BMI categories

The risk for HF hospitalization significantly differed across sex-BMI ≥ 25 kg/m^2^ categories (overall *p*-value < 0.001). However, the two comparisons of interest (women vs. men across BMI groups) were not significant. The IRRs for women versus men when BMI < 25 kg/m^2^ and ≥ 25 kg/m^2^ were 0.78, 95% CI = 0.59–1.04; *p* = 0.092, and 1.08, 95% CI = 0.92–1.28; *p* = 0.354, respectively.

### sNEP and risk of HF hospitalization

**Figure 1:**
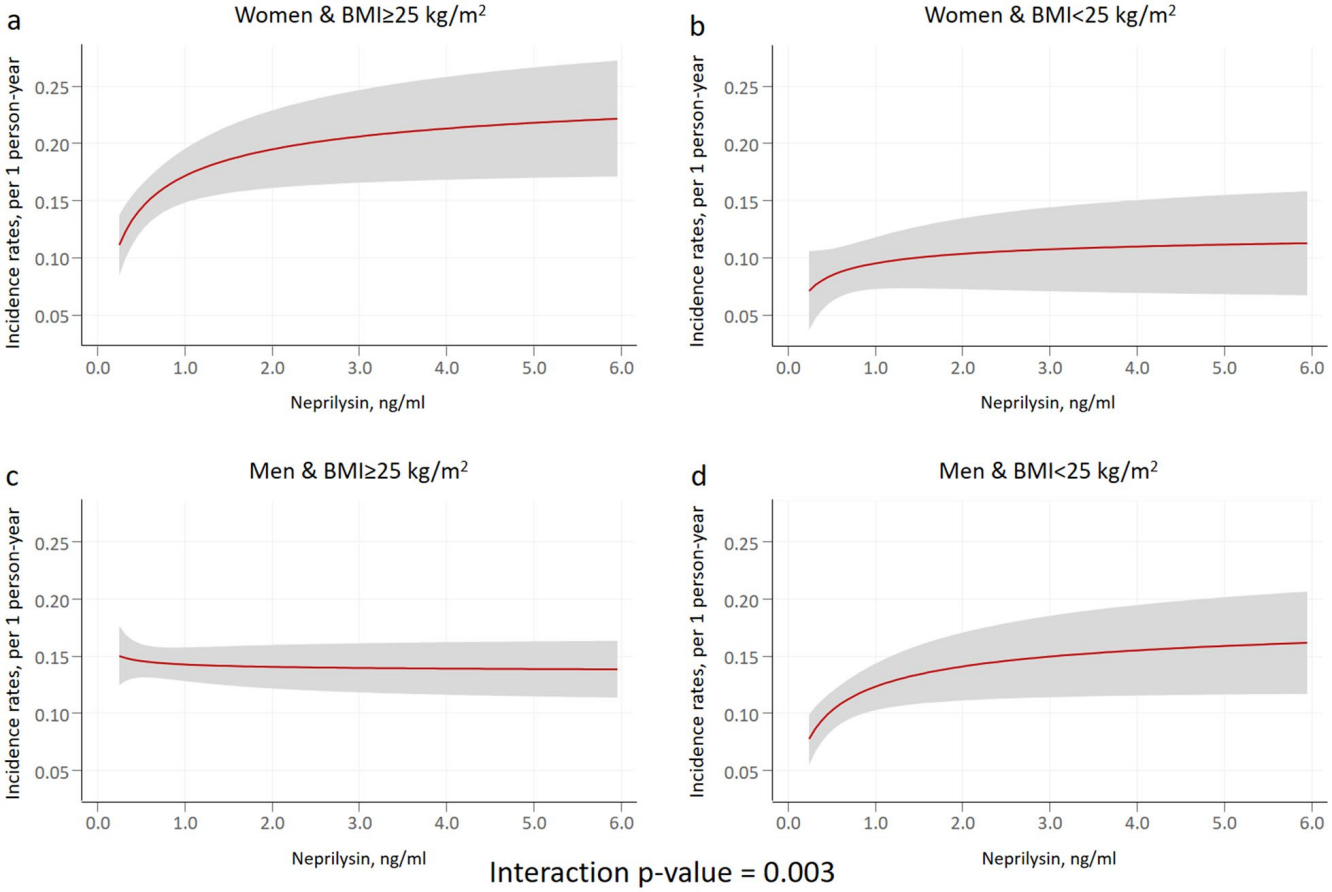
Soluble neprilysin and heart failure admission rates among sex and body mass index categories. Estimates were adjusted for age, HF duration, BMI, NYHA III versus I–II, ischaemic heart disease, systolic blood pressure, heart rate, eGFR, sodium, high-sensitivity troponin T, LVEF, and treatment with ACEI/ARB, beta-blockers, MRA, or loop diuretics. ACEI: angiotensin-converting enzyme inhibitors; ARB: angiotensin II receptor blockers; BMI: body mass index; eGFR: estimated glomerular filtration rate; HF: heart failure; LVEF: left ventricle ejection fraction; MRA: mineralocorticoid receptor antagonist; NT-proBNP: amino-terminal pro-B-type natriuretic peptide; NYHA: New York Heart Association.

**Figure 2:**
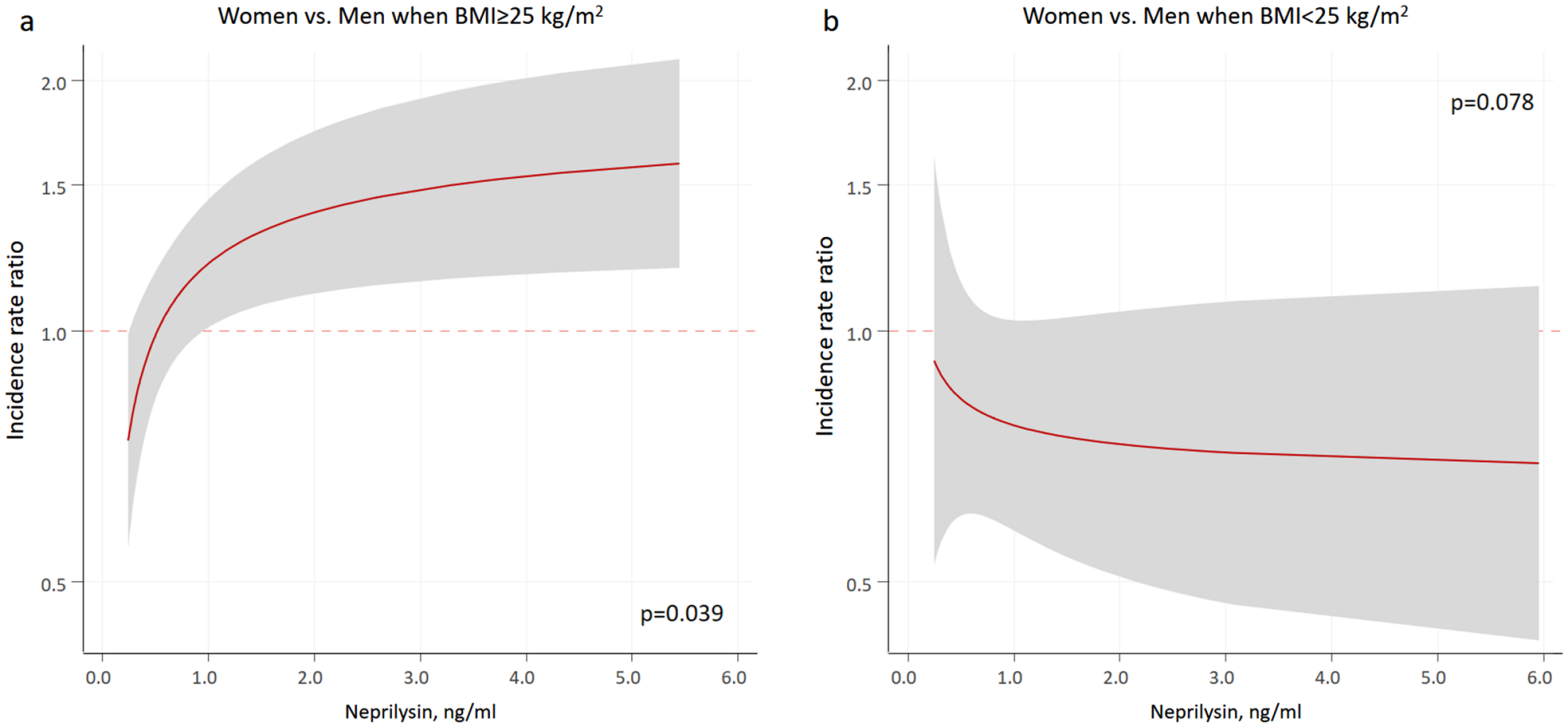
Soluble neprilysin and excess hospitalization risk in women versus men across body mass index values. BMI: body mass index.

sNEP was a significant predictor of HF hospitalizations, either when modeled (as the main effect) continuously [FP-0.5] (*p* = 0.001) or in quintiles (omnibus *p* = 0.016). Compared to patients in the lower sNEP quintile, patients in the upper quintile had an increased risk of HF hospitalizations (IRR = 1.29; 95% CI: 1.06–1.58; *p* = 0.013). The incidence rates of this endpoint along the continuum of sNEP across the evaluated subgroups are shown in Fig. 1. The interaction analysis revealed a differential prognostic effect of sNEP (modeled as continuous with FP [-0.5]) across sex and BMI categories for HF hospitalizations (*p*-value for interaction = 0.003). Indeed, the sNEP trajectory showed a positive and non-linear association in women with BMI ≥ 25 kg/m^2^ compared to the men counterpart (*p* = 0.039) (Figs. 1 and 2a). Differences in gender were not present when BMI < 25 kg/m^2^ (*p* = 0.078) (Figs. 1 and 2b). Table 3 shows the estimates of risk (women vs. men) for different values of sNEP across BMI status.

**Table 3:** sNEP and adjusted risk of adverse outcomes across sex and BMI.

	BMI ≥ 25 kg/m^2^		BMI < 25 kg/m^2^	
	Women vs. men		Women vs. men	
	IRR	95% CI	IRR	95% CI
**HF-hospitalizations**				
0.25 ng/mL^a^	0.78	0.60–1.02	0.91	0.53–1.56
0.39 ng/mL^b^	0.91	0.74–1.11	0.85	0.59–1.24
0.64 ng/mL^c^	1.06	0.90–1.26	0.80	0.60–1.07
1.22 ng/mL^d^	1.26	1.05–1.53	0.76	0.56–1.03
3.24 ng/mL^e^	1.50	1.16–1.94	0.71	0.47–1.10
**All-cause mortality**				
0.25 ng/mL^a^	0.62	0.44–0.84	0.90	0.63–1.41
0.39 ng/mL^b^	0.71	0.57–0.90	0.84	0.59–1.13
0.64 ng/mL^c^	0.83	0.68–1.02	0.80	0.62–1.03
1.22 ng/mL^d^	0.97	0.76–1.22	0.78	0.54–1.06
3.24 ng/mL^e^	1.12	0.81–1.52	0.72	0.40–1.11

*BMI* body mass index, *sNEP* serum neprilysin.

^a^Percentile 10% of sNEP distribution.

^b^Percentile 25% of sNEP distribution.

^c^Percentile 50% of sNEP distribution.

^d^Percentile 75% of sNEP distribution.

^e^Percentile 90% of sNEP distribution.

A subgroup analysis stratifying the samples across LVEF (≤ 40% and > 40%), with the same multivariate setting, suggests that the direction and magnitude of the excess risk attributable to higher sNEP in women with BMI ≥ 25 kg/m^2^ were found in both subgroups (Supplementary Fig. 1).

In a sensitivity analysis, we confirmed the differential sNEP gradient of risk across sex and BMI strata when BMI was categorized by the median value (Supplementary Fig. 2).

### sNEP and mortality risk—interaction analysis

**Figure 3:**
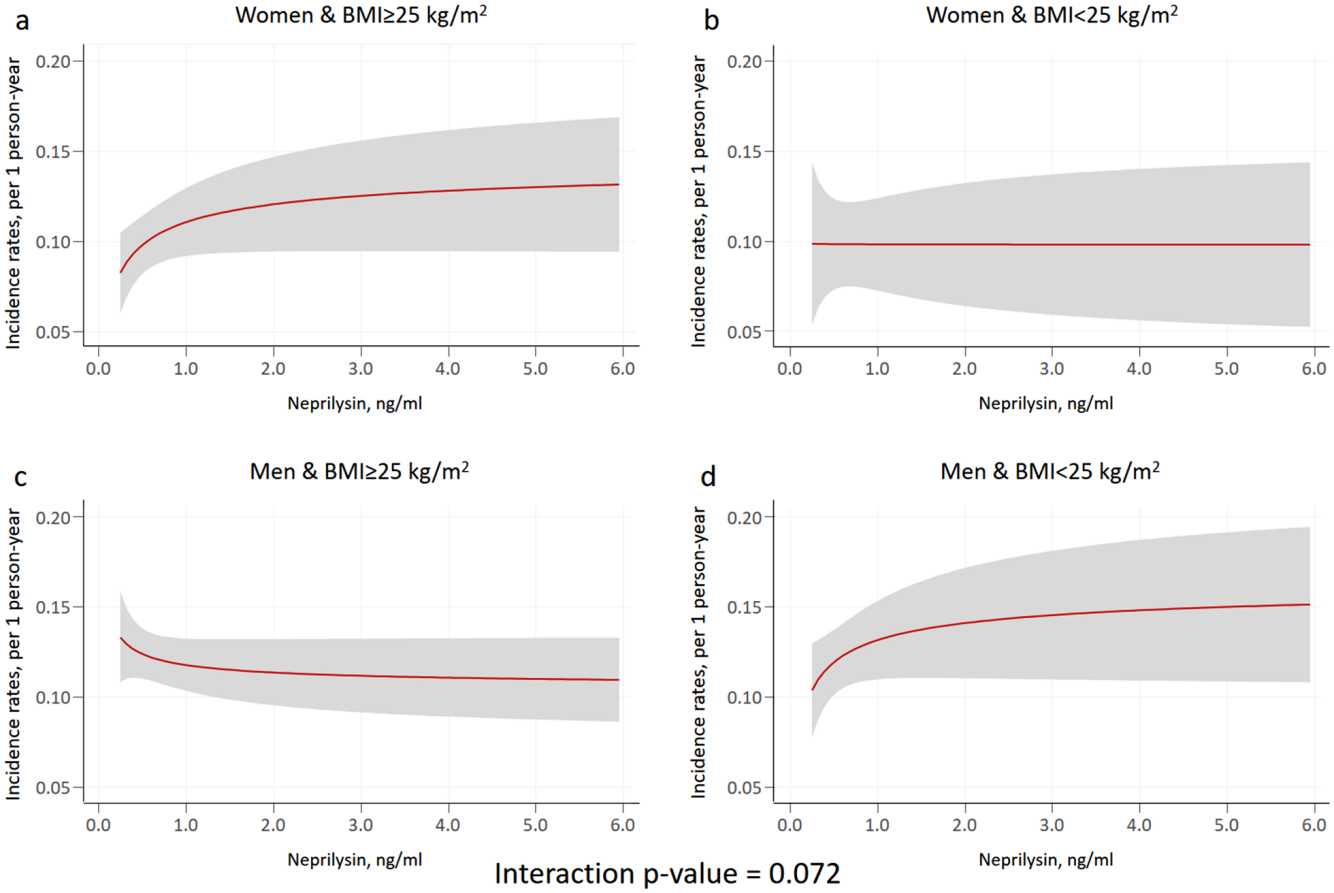
Soluble neprilysin and mortality rates among sex and body mass index categories. Estimates of risk were adjusted for age, HF duration, BMI, NYHA III versus I–II, ischaemic heart disease, systolic blood pressure, heart rate, eGFR, sodium, high-sensitivity troponin T, haemoglobin, ST2, LVEF, and treatment with ACEI/ARB, beta-blockers, MRA, or loop diuretics. ACEI: angiotensin-converting enzyme inhibitors; ARB: angiotensin II receptor blockers; BMI: body mass index; eGFR: estimated glomerular filtration rate; HF: heart failure; LVEF: left ventricle ejection fraction; MRA: mineralocorticoid receptor antagonist; NYHA: New York Heart Association.

**Figure 4:**
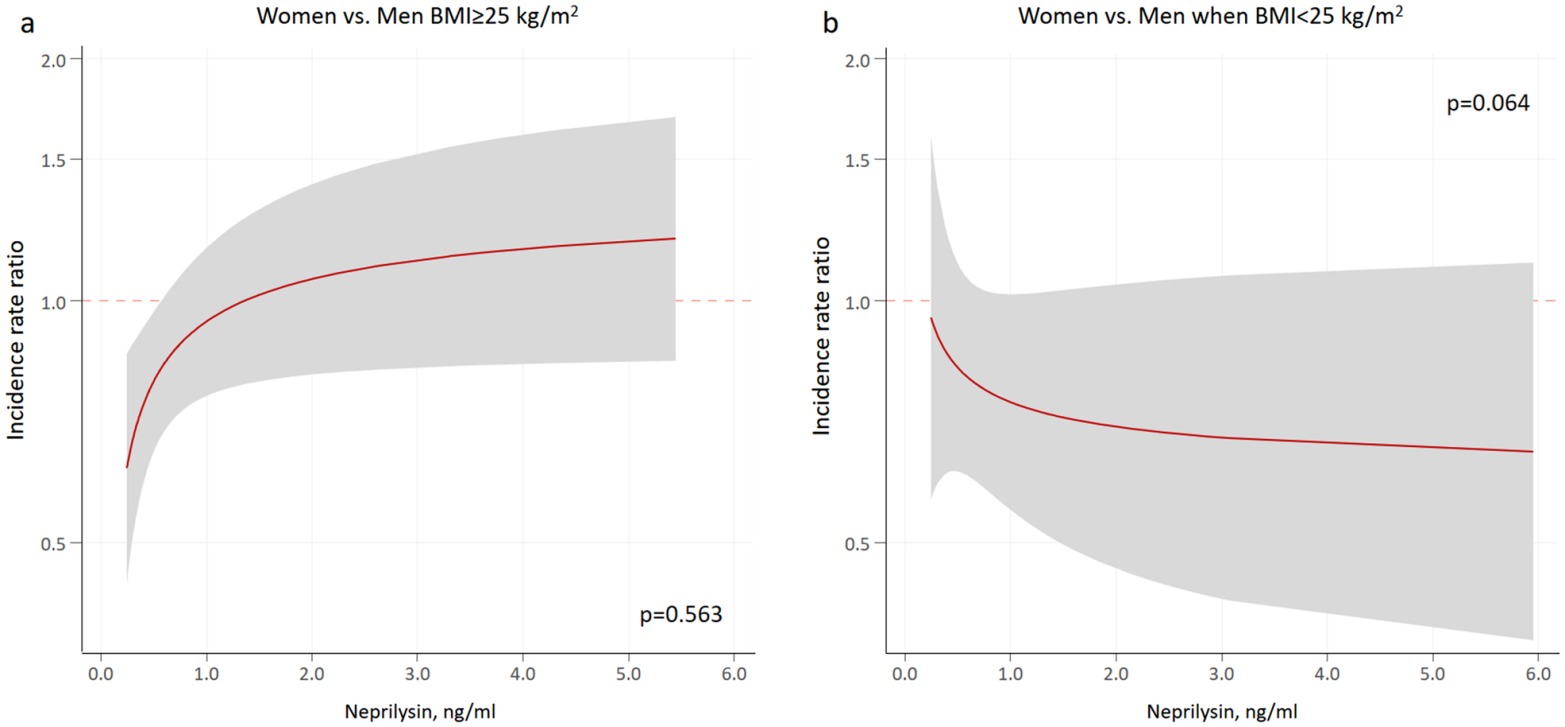
Soluble neprilysin and excess mortality risk in women versus men across body mass index values. BMI: body mass index.

In multivariable analysis, sNEP (main effect) (modeled as continuous with FP [-0.5]) was not associated with mortality risk (*p* = 0.241). The prognostic effect of sNEP on mortality did not significantly differ across sex and BMI categories (*p*-value for interaction = 0.072; Fig. 3). Indeed, any of the two comparisons of interest (women vs. men in the two BMI groups (BMI ≥ 25 kg/m^2^ vs. BMI < 25 kg/m^2^) were not significant (*p* = 0.563 and *p* = 0.064, respectively) (Figs. 4a, b). Worth noting, however, is that the sNEP depicted trajectories are almost identical to those found for HF hospitalization. The estimates of risk (women vs. men) for different values of sNEP are presented in Table 3.

## Discussion

In this large cohort of ambulatory patients with chronic HF and predominantly reduced LVEF, sNEP predicted the risk of HF hospitalization, especially in overweight women. Our data suggested that sNEP activity may be influenced by sex and excess adipose tissue.

The following explanations have been postulated to backed-up such interaction. Mature adipocytes are known to produce and express neprilysin^16^, and obese individuals have increased levels of sNEP in proportion to their body mass^17^. In the setting of HF, a leptin-aldosterone-neprilysin axis has been proposed^7^. Packer postulated that obesity aggravates the deleterious interaction of leptin with the renin–angiotensin–aldosterone system and the renal sympathetic system leading to overactivity of neprilysin and, thus, deficiency in endogenous natriuretic peptides in these patients^7^. Also, it has been postulated that obesity-driven aldosterone and neprilysin overactivity generate a vicious circle that further stimulates adipogenesis, adipocyte dysfunction, and adipose tissue inflammation, thereby enhancing this deleterious feedback loop^7,18^.

There is no clear explanation about the positive association between sNEP and the risk of HF hospitalizations in overweight women, particularly in the absence of significantly elevated levels of sNEP in this subgroup. Complicating this issue is also the controversy about the utility of sNEP as a proxy of NEP activity^7^. We speculate that sNEP may better reflect NEP activity in the subset of overweight women, a hypothesis that is endorsed by some findings regarding lower medians of NT-proBNP in this group. On this same line, we recently proposed the concept of "natriuretic peptide availability" based on the balance between natriuretic peptide synthesis and degradation (which is mediated by neprilysin)^19^. Elaborating upon this concept, the neprilysin/NT-proBNP ratio (or alternative formulas integrating both biomarkers) could serve as an alternative surrogate for neprilysin activity, which would be particularly useful in obese patients especially given the complexity of measuring neprilysin activity itself.

The clinical association between sNEP and sex reported here, and in the PARAGON-HF trial of patients with HF and preserved ejection fraction^4^, raises the question as to whether these findings reflect association or causation^20^. Following the framework of the leptin-aldosterone-neprilysin axis, Packer postulated that women and the elderly have high levels of aldosterone, leptin, and neprilysin, with lower endogenous natriuretic peptide levels^7^. A biological difference cannot be ruled out, as oestrogens have been reported to up-regulate the expression of neprilysin^21^.

Nevertheless, most patients enrolled in HF studies, including ours, are post-menopausal women; as such, oestrogen deficiency would yield low sNEP levels. We humbly acknowledge the possibility of dark corners in the complex regulation and counter-regulation of the neuro-hormonal axis in HF that escape our understanding. Importantly, the findings observed in the setting of HF do not concur with those of the general population, not affected by neurohormonal activation. A recent report of 1536 participants from Olmsted County, Minnesota, did not find significant associations between sNEP levels and age, sex, or renal function^12^.

### Study limitations

The present study has some limitations. First, our study included a Mediterranean cohort of predominantly white men and reduced systolic function HF patients, and thereby limiting the extrapolation of our results to other ethnicities or patients with HF and preserved ejection fraction. Also, the proportion of obese patients in this cohort precluded exploring whether this sex-BMI differential effect of sNEP may be even greater in obese patients. Second, analytical issues regarding sNEP measurement must be resolved before its routine implementation in daily clinical practice. Third, the lack of serial assessment of sNEP and BMI status during the follow-up precluded inferring how the association here found may describe a dynamic pattern. Fourth, the low proportion of patients/events in some comparisons, especially in patients with LVEF ≥ 40%, decreased the statistical power and increasing the possibility of a type II error. Finally, with the present data, we cannot explore the potential role of sNEP for tailoring sacubitril/valsartan.

## Conclusions

In chronic HF with predominant left systolic dysfunction, high sNEP levels in overweight women predicted an increased risk for recurrent HF hospitalizations. Further studies are warranted to explore whether sNEP levels can be used to tailor angiotensin receptor blocker and neprilysin inhibitor therapy. The more knowledge we gain in HF's pathobiology and its management, the greater the need for a precision medicine approach centered on disease biology with biomarkers as surrogates.

## Supplementary Information

Supplementary Information 1.

Supplementary Information 2.
