# Supplementary figures and images for: The influence of sex and body mass index on the association between soluble neprilysin and risk of heart failure hospitalizations
Source: Sci Rep. 2021 Mar 15;11:5940. doi: 10.1038/s41598-021-85490-1 (PMC7960699; doi:10.1038/s41598-021-85490-1)

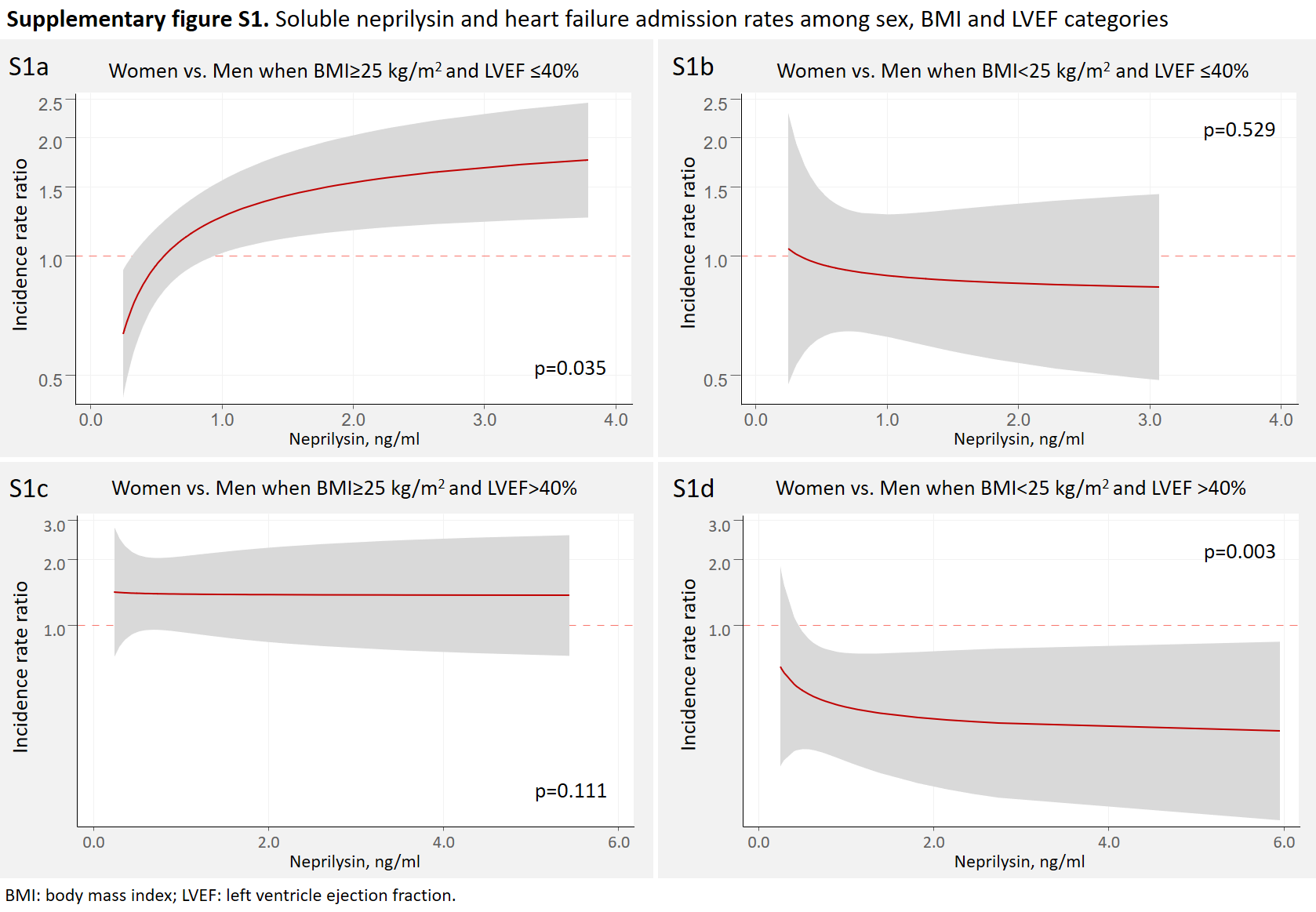

Supplement: Supplementary file 1 — Supplementary Information 1. [file 41598_2021_85490_MOESM1_ESM.tif]

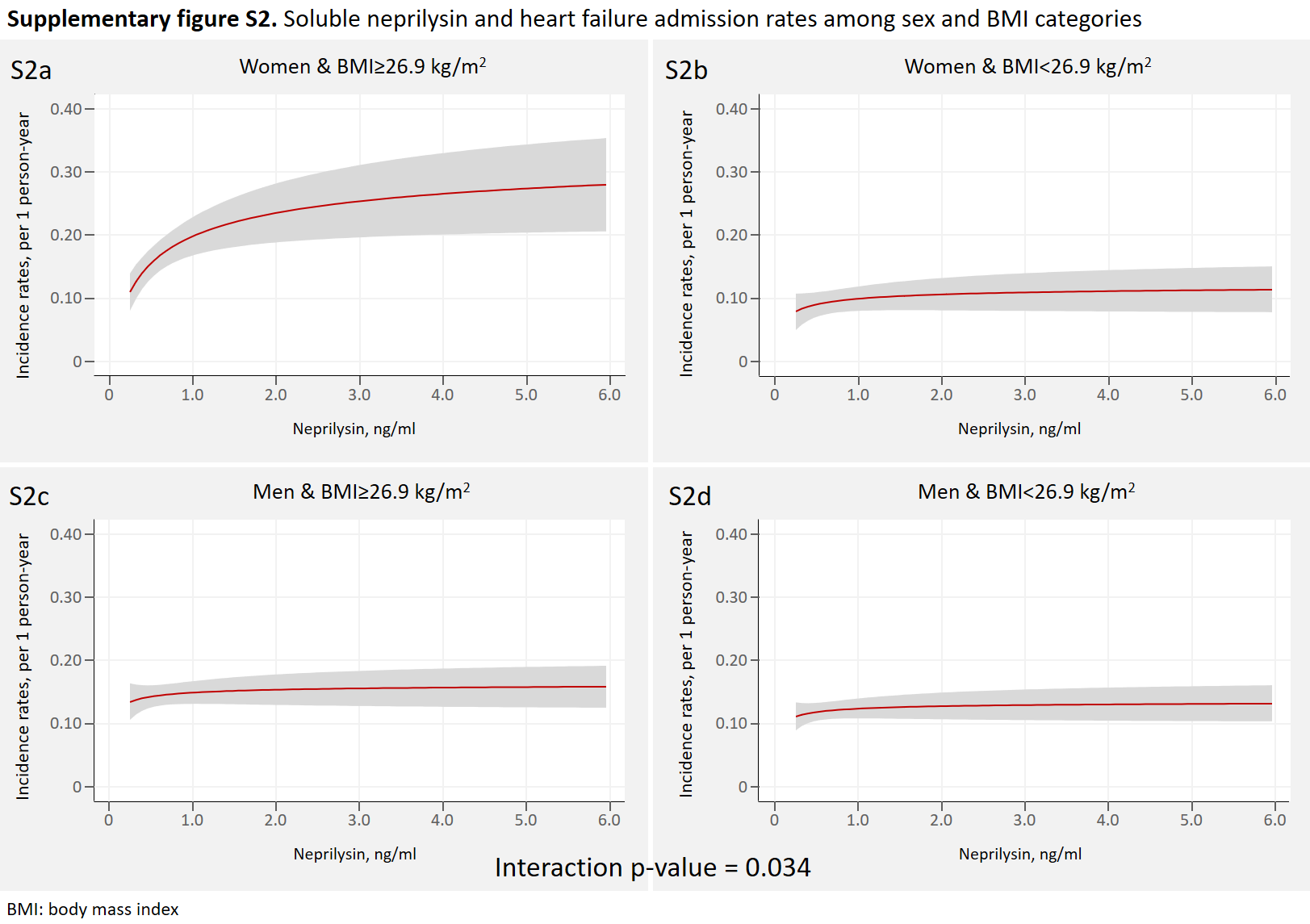

Supplement: Supplementary file 2 — Supplementary Information 2. [file 41598_2021_85490_MOESM2_ESM.tif]
